# Advanced glycation end products (AGE) and receptor for AGE (RAGE) in patients with active tuberculosis, and their relationship between food intake and nutritional status

**DOI:** 10.1371/journal.pone.0213991

**Published:** 2019-03-14

**Authors:** Lívia Fontes da Silva, Erika Cavalheiro Skupien, Tássia Kirchmann Lazzari, Sizuane Rieger Holler, Ellis Gabriela Correa de Almeida, Luísa Rebechi Zampieri, Sandra Eugênia Coutinho, Michael Andrades, Denise Rossato Silva

**Affiliations:** 1 Programa de Pós-Graduação em Ciências Pneumológicas, Universidade Federal do Rio Grande do Sul, Porto Alegre, Brazil; 2 Faculdade de Medicina, Universidade Federal do Rio Grande do Sul, Porto Alegre, Brazil; 3 Faculdade de Nutrição, Universidade Federal do Rio Grande do Sul, Porto Alegre, Brazil; 4 Hospital de Clínicas de Porto Alegre, Porto Alegre, Brazil; Karolinska Institutet, SWEDEN

## Abstract

**Introduction:**

The receptor for advanced glycation end products (RAGE) is expressed in normal lungs and is upregulated during infection. AGEs and RAGE cause oxidative stress and apoptosis in lung cells. The objective of this study is to evaluate levels of AGEs and its soluble receptor (sRAGE), and to investigate their relationship with food intake and nutritional status, in a university-affiliated hospital in Brazil.

**Methods:**

Case-control study, from June 2017 to June 2018. AGE (carboxymethyl lysine, CML) and sRAGE were measured from blood samples by Elisa. Nutritional assessment was performed by body mass index, triceps skin-fold thickness, mid-arm circumference, mid-arm muscle circumference, bioelectrical impedance analysis, and food frequency questionnaire.

**Results:**

We included in the study 35 tuberculosis (TB) patients and 35 controls. The mean sRAGE levels were higher in TB patients than in controls (68.5 ± 28.1 vs 57.5 ± 24.0 pg/mL; p = 0.046). Among cases that were current smokers, lower sRAGE levels were associated with mortality, evaluated at the end of hospitalization (p = 0.006), and with weight loss (p = 0.034). There was no statistically significant difference in CML levels and diet CML content between cases and controls. Malnutrition was more frequent in cases, but there was no correlation between nutritional parameters and CML or sRAGE levels.

**Conclusions:**

TB patients had higher sRAGE levels than controls, although it is not clear that this difference is clinically relevant. Also, sRAGE was associated with weight loss and mortality.

## Introduction

Tuberculosis (TB) is a major public health problem worldwide, especially in low- and middle-income countries, and it is caused by *Mycobacterium tuberculosis* complex, which includes: *Mycobacterium tuberculosis* (TB in humans); *M*. *africanum* (TB in humans only in certain regions of Africa); *M*. *microti* (TB only in voles); *M*. *bovis*, *M*. *caprae* and *M*. *pinnipedii* (TB in wild and domesticated mammals). It is estimated that one-third of the world’s population is infected with Mycobacterium tuberculosis, and 8 million develop the active form of the disease each year, resulting in 2 million deaths per year.[1] Brazil is in 18th place among the 22 countries responsible for 80% of TB cases globally, with a cumulative incidence of 32.4 cases / 100,000 inhabitants in 2016.[2].

The pathogenesis of the consumptive syndrome, which is long recognized as a characteristic of TB, is largely unknown. The proinflammatory cytokines are the initial candidates as agents causing the metabolic alterations that eventually result in the consumptive TB syndrome.[3] In addition to the pro-inflammatory cytokines, cell-mediated immunity and innate immune responses play an important role in the host response to mycobacterial infection, contributing to disease severity and complications in active TB.[4,5].

The receptor for advanced glycation end products (RAGE) is expressed in normal lungs and is upregulated during inflammation and infection.[6–9] RAGE is a pattern-recognition receptor that binds multiple ligands, like amyloid beta (Aβ), high-mobility group box 1 (HMGB1), lipopolysaccharide (LPS), macrophage-1 antigen (Mac-1), phosphatidylserine, S100A12, and AGEs.[10–15]. AGEs are a heterogeneous group of irreversible products resulting from nonenzymatic glycation between reducing sugars and free amino groups of proteins, nucleic acids, or lipids.[15,16] The common AGEs in foods and human plasma include pentosidine, carboxymethyl lysine (CML) and furosine, and CML has been considered the predominant AGE in human plasma. [17] The interaction between AGEs and RAGE on the plasma membrane causes inflammation, oxidative stress, and apoptosis in lung cells.[18] One study demonstrated that RAGE deficient mice displayed more body weight loss and enhanced mortality.[19] However, studies investigating the relationship between food intake, nutritional status, AGE and RAGE levels and TB, are mostly with animal models.[19,20] Thus, the objective of this pilot study is to evaluate AGEs and RAGE levels in patients with active TB and healthy controls, and to investigate the relationship between food intake and nutritional status with AGEs and RAGE levels.

## Material and methods

### Study design and location

We conducted a prospective case-control study in a general, tertiary care, university-affiliated hospital (Hospital de Clínicas de Porto Alegre–HCPA), from June 2017 to June 2018. TB patients and controls were individually matched for sex and age in a 1:1 matching ratio. Patients were recruited at HCPA inpatient’s units. The control group consisted of volunteers recruited in the same hospital, selected among healthy members of the patients’ family (who were accompanying the patients at the hospital). We decided this because cohabitants are exposed to the same risk factors for tuberculosis and are likely to have a similar diet (important because of the dCML assessment). In Brazil, like in many other places, family members who cohabit with patients with TB were examined to exclude active TB and to detect latent TB. If family member have active or latent TB, he/she was not included in the study. The study was conducted according to the Declaration of Helsinki, was approved by the Ethics Committee at HCPA (number 14–0044), and all subjects gave written informed consent to participate.

### Patients and data collection

Patients with a confirmed diagnosis of TB, older than 18 years, who agreed to participate, were included in the study. We excluded patients with extrapulmonary TB, and those who had been on pulmonary TB treatment for more than 3 days, and patients and controls who were not able to perform the study procedures, diabetics, pregnant women and those with a history of previous TB.

After signing informed written consent enrolled subjects were interviewed using a standardized questionnaire. The following data were recorded: demographic data (sex, age, race, years of schooling), presence of cough, fever, night sweating, hemoptysis, sputum production, weight loss, dyspnea, chest pain, smoking status, alcohol consumption, drug use, presence of comorbidities. Smoking and alcohol abuse were defined as described in detail previously. [21] We also recorded the results of the main diagnostic tests performed, as well as the outcome of hospitalization (discharge or death). An independent physician analyzed the chest X-rays and classified them as typical or compatible with active TB, according to previously described guidelines.[22] The diagnosis of pulmonary TB was based on consensus criteria.[23].

### Nutritional assessment

Nutritional assessment was performed by body mass index (BMI), triceps skin-fold thickness (TSF), mid-arm circumference (MUAC), mid-arm muscle circumference (MAMC), bioelectrical impedance analysis (BIA), and food frequency questionnaire (FFQ), as described in detail previously [21].

### Laboratory tests

Blood sample was collected after an overnight fast. After collection, the blood was centrifuged and frozen at -80°C. CML has been considered the predominant AGE in human plasma (86%) [24], and for that reason we have chosen to measure it. CML and RAGE were measured by Elisa, in duplicate, according to the manufacturer’s instructions (CML: OxiSelect N-epsilon-(Carboxymethyl) Lysine [Cell Biolabs Inc, San Diego, CA]; RAGE: Human RAGE Quantikine [R&D Systems Inc., Minneapolis, MN]).

### Statistical analysis

Data analysis was performed using IBM SPSS Statistics for Windows, version 22.0 (Armonk, NY, IBM Corp). Data were presented as number of cases, mean ± standard deviation (SD), or median with interquartile range (IQR). Shapiro-Wilk test was used for testing normality. Categorical comparisons were carried out by chi-square test (McNemar). Continuous variables were compared using the paired *t*-test or Wilcoxon test. Pearson’s (or Spearman’s when indicated) correlations was performed to evaluate for potential relationships. A two-sided p value ≤ 0.05 was considered significant for all analyses. There is only one study [19] that evaluated the association of mortality and RAGE; however, this study was conducted in TB animal models, which makes it difficult to use for the calculation of sample size in human studies. Then, the sample size was calculated based on the expected difference of sRAGE levels between survivors and non-survivors of 15 pg/mL. Considering a confidence level of 95% and a power of 80%, we estimated a sample of 30 individuals per group.

## Results

During the study period, 35 TB patients and 35 controls met the inclusion criteria and were included in the analysis. Among TB cases, the most frequent symptoms were: weight loss (94.3%, n = 33), cough (88.6%, n = 31), night sweats (65.7%, n = 23), and fever (62.9%, n = 22). White race was more common among controls than in cases (82.9% vs 54.3%; p = 0.02). Among TB cases, fourteen patients (40.0%) were HIV positive, 28 (80.0%) were smear positive (sputum smear-positive TB based on the presence of at least one acid-fast bacillus in at least one sputum sample), and 28 (80.0%) had a positive culture (culture positive for Mycobacterium tuberculosis complex). Twenty-six patients (74.3%) had a typical chest X-ray, and 9 (25.7%) had a compatible chest X-ray. Among TB patients, there were 28 (80%) survivors and 7 (20%) non-survivors.

Table 1 shows the comparison between cases and controls. The mean sRAGE levels were higher in TB patients than in controls [68.5 ± 28.1 pg/mL vs 57.5 ± 24.0 pg/mL, p = 0.046]. There was no statistically significant difference in CML levels and diet CML content between cases and controls. BMI was significantly lower in cases than in controls (p<0.0001). Also, undernutrition by MUAC, MAMC, and TSF were more frequent in cases than in controls.

**Table 1:** Comparison between cases and controls.

Variables	Cases n = 35	Controls n = 35	p value
Male sex, n (%)	24 (68.6)	24 (68.6)	-
Age (years), mean ± SD	37.5 ± 16.7	38.6 ± 16.4	0.768**
White race, n (%)	19 (54.3)	29 (82.9)	0.02*
Current smoking, n (%)	22 (62.9)	16 (45.7)	0.23*
Alcohol abuse, n (%)	13 (37.1)	7 (20.0)	0.186*
Drug use, n (%)	15 (34.3)	7 (20.0)	0.282*
HIV positive, n (%)	14 (40.0)	3 (8.6)	0.005*
Systemic arterial hypertension, n (%)	0	3 (8.6)	0.52*
BMI (kg/m^2^), mean ± SD	19.5 ± 3.6	26.9 ± 4.9	<0.0001**
Body fat (%, BIA), mean ± SD	23.1 ± 10.2	24.3 ± 8.9	0.60**
Reduced body fat (BIA), n (%)	5 (14.3)	3 (8.6)	0.71*
Malnutrition by MUAC, n (%)	16 (45.7)	4 (11.4)	0.001*
Malnutrition by MAMC, n (%)	17 (48.6)	9 (25.7)	0.04*
Malnutrition by TSF, n (%)	13 (37.1)	3 (8.6)	0.004*
Diet CML content (kU/day), median (IQR)	1.29 (0.62–1.81)	1.35 (1.07–2.13)	0.243***
Serum CML (μg/μL), median (IQR)	0.06 (0.03–0.10)	0.07 (0.03–0.14)	0.453***
sRAGE (pg/mL), mean ± SD	68.5 ± 28.1	57.5 ± 24.0	0.046**

*Chi-square test (McNemar).

** Paired t-test.

*** Wilcoxon test

SD: standard deviation. BMI: body mass index. BIA: bioelectrical impedance analysis. IQR: interquartile range. MUAC: Mid-upper arm circumference. MAMC: Mid-arm muscle circumference. TSF: Triceps skin-fold. AGE: advanced glyucation end products. CML: carboxymethyl lysine. sRAGE: soluble receptor of advanced glycation end products.

There was no statistically significant correlation between serum CML levels and diet CML content (r = -0.13; p = 0.33).Also, there was no significant correlation between BMI and CML levels (r = -0.15; p = 0.24), and between BMI and sRAGE levels (r = -0.11; p = 0.39). TSF, MUAC, and MAMC were also not correlated with CML or sRAGE levels. Diet CML content was not different between cases and controls (p = 0.243).

There was no statistical difference in serum CML levels, diet CML content and sRAGE levels between survivors and non-survivors (p = 0.771, p = 0.191 and p = 0.163, respectively). However, among cases that were current smokers, lower sRAGE levels were associated with mortality (sRAGE levels [mean ± SD] = 58.0 ± 36.5 pg/mL [non-survivors] vs 71.3 ± 25.6 pg/mL [survivors], p = 0.006; odds ratio 1.053 [CI95% 1.006–1.103]). In addition, among cases, lower sRAGE levels were associated with weight loss (sRAGE levels [mean ± SD] = 65.6 ± 27.4 pg/mL [weight loss] vs 98.6 ± 16.7 pg/mL [no weight loss], p = 0.034).

## Discussion

In this case-control study, we aimed to evaluate AGEs and RAGE levels in patients with active TB and healthy controls, and the relationship between food intake and nutritional status with AGEs and RAGE levels. This was a pilot study, much more hypothesis-generating than designed to answer key mechanistic questions on the roles of RAGE in tuberculosis and the use of sRAGE as a reliable prognostic tool in tuberculosis. We found that sRAGE levels were higher in TB patients than in controls. Among cases that were current smokers, lower sRAGE levels were associated with weight loss and mortality. In addition, we did not observe a correlation between serum CML levels and diet CML levels. Also, we identified high prevalence of undernutrition in TB cases, regardless of the measurement used to assess nutritional status. However, there was no significant correlation between those parameters and CML or sRAGE levels.

RAGE is a cell-surface receptor belonging to the immunoglobulin superfamily.[25] There is evidence of ligand/RAGE signaling pathway activation in a wide spectrum of diseases, including diabetes mellitus, cancer, chronic renal failure, and rheumatoid arthritis.[26] RAGE is expressed at low levels in normal lung and becomes upregulated in conditions associated with inflammation and lung damage.[6–9] Therefore, RAGE’s inflammatory pathway is not specific of a single lung disease. RAGE overexpression has been described in smoke-related pulmonary disease, postobstructive pneumonia, organizing pneumonia, granulomatous disease, and usual interstitial pneumonia.[7].

We found higher sRAGE levels in TB patients as compared with controls, although it is not clear that this difference is clinically relevant. sRAGE functions as a ligand-binding decoy, a competitive inhibitor of RAGE, protecting sensitive cells from the potentially deleterious effects of their hyperactivity.[27] Reduced sRAGE levels have been observed in a number of chronic diseases such as diabetes mellitus, chronic renal failure, and cancer.[28] On the other hand, in accordance with our findings, Watanabe et al[29] reported elevated levels of sRAGE in asthmatic sputum, and Uchida et al.[8] report significantly higher sRAGE levels both in pulmonary edema fluid, and in plasma from patients with ALI/ARDS (acute lung injury/acute respiratory distress syndrome) compared to healthy volunteers. A possible explanation may be that enhanced RAGE expression and cellular damage might increase sRAGE generation and release. In this context, sRAGE levels could reflect RAGE hyperactivity.[26].

In contrast, lower sRAGE levels were associated with weight loss and mortality in the present study, but only among cases that were current smokers. Similar findings were described by van Zoelen et al[19] in murine models. The authors demonstrated that pulmonary RAGE expression was increased during TB, and that RAGE deficient mice displayed more body weight loss and enhanced mortality. In addition, plasma levels of sRAGE were previously described to be significantly lower in smokers.[30].

CML is the predominant AGE in human plasma, and is over synthesized in conditions associated with inflammation and lung damage.[7] However, there was no statistically significant difference in CML levels between cases and controls in the present study. In fact, the small sample size could prevent us from finding differences. Nevertheless, there may be another explanation. RAGE is a pattern-recognition receptor that binds multiple ligands, not only AGEs, but also Aβ, HMGB1, LPS, Mac-1, phosphatidylserine and S100A12.[10–15]. Indeed, one study[31] showed that HMGB1/RAGE signaling may play an important role in pathogenesis and disease manifestations in non-HIV adults with active pulmonary TB. In this investigation, RAGE and HMGB1 gene expressions correlated positively with clinic-radiodiological severity.

Undernutrition is common among patients with TB[32,33] and is associated with disease severity and unfavorable outcomes.[34,35]. In our sample, approximately 40% of TB patients had criteria for undernutrition, according to BMI, TSF, MUAC and MAMC. Nonetheless, there was no significant correlation between any of these measurement and CML or sRAGE levels.

Our study has some limitations. First, the investigation was done in a single center. However, we assume that there is no reason why these results do not apply to other settings. Also, the sRAGE levels are low in both cases and controls when compared to previously published articles, and this could decrease the external validity of the current findings. In addition, we don’t know the significance of the difference of sRAGE level of 15pg/ml between survivors and non-survivors chosen to calculate sample size. The clinical relevance of the observed difference in sRAGE between cases and controls is undetermined, and should be tested in larger studies. Still, the higher prevalence of HIV among cases could have been a confounding factor resulting in the observed differences of sRAGE levels between cases and controls. Also, we estimated daily dietary CML content from a FFQ and not from 3-day food records, probably the most adequate method. More important, the dietary AGE database that we used included foods selected from diets common in northeastern US area, and may thus not represent Brazilian diet. In spite of these concerns, the strength of the present study is that it is the first pilot study to generate a hypothesis on the role of CML/RAGE in TB patients and not in animal models.

In conclusion, in this pilot study, we demonstrated higher sRAGE levels in TB patients in comparison with controls. At least among cases that were current smokers, lower sRAGE levels were associated with weight loss and mortality. Future studies with larger sample size with a 1:2 or 1:3 matching are necessary to confirm these findings. Additionally, researches focusing on other ligand/RAGE signaling pathways may provide a better understanding of immunopathogenic processes involved in TB.
